# Automation in the Life Science Research Laboratory

**DOI:** 10.3389/fbioe.2020.571777

**Published:** 2020-11-13

**Authors:** Ian Holland, Jamie A. Davies

**Affiliations:** Deanery of Biomedical Science and Synthsys Centre for Synthetic and Systems Biology, University of Edinburgh, Edinburgh, United Kingdom

**Keywords:** laboratory automation, life science research, automation design, research efficiency, reproducibility, innovation inhibition, environmental design

## Abstract

Protocols in the academic life science laboratory are heavily reliant on the manual manipulation of tools, reagents and instruments by a host of research staff and students. In contrast to industrial and clinical laboratory environments, the usage of automation to augment or replace manual tasks is limited. Causes of this ‘automation gap’ are unique to academic research, with rigid short-term funding structures, high levels of protocol variability and a benevolent culture of investment in people over equipment. Automation, however, can bestow multiple benefits through improvements in reproducibility, researcher efficiency, clinical translation, and safety. Less immediately obvious are the accompanying limitations, including obsolescence and an inhibitory effect on the freedom to innovate. Growing the range of automation options suitable for research laboratories will require more flexible, modular and cheaper designs. Academic and commercial developers of automation will increasingly need to design with an environmental awareness and an understanding that large high-tech robotic solutions may not be appropriate for laboratories with constrained financial and spatial resources. To fully exploit the potential of laboratory automation, future generations of scientists will require both engineering and biology skills. Automation in the research laboratory is likely to be an increasingly critical component of future research programs and will continue the trend of combining engineering and science expertise together to answer novel research questions.

## Introduction

The progressive integration of automation into work environments has enhanced the production rates, efficiency and quality of an enormous array of industrial processes (Hitomi, 1994; Autor, 2015). From generation to generation, mechanised tooling has replaced swathes of manual tasks. More recent advances in robotics and information technology have further automated processes that were once the sole domain of human brawn or brain (Hasegawa, 2009). Life science research conducted within academic institutions has also welcomed the ingress of mechanised equipment designed to automate a range of tasks. However, it is noticeable that a typical university research laboratory, often led by a single principal investigator, maintains a high level of manual manipulation in the form of undergraduate, postgraduate, post-doctoral and technical staff. Many experimental procedures remain heavily reliant upon the individual researcher manually carrying out protocols at the research bench.

This is in contrast to industrial environments, where widespread investment in automation has allowed companies to maximise their outputs and increase profits (Ravazzi and Villa, 2009). Laboratories in a clinical setting have also experienced the benefits of adopting automation (Hawker et al., 2018), increasing the speed and reliability of patient-specific data for use by clinicians (Sarkozi et al., 2003; Lou et al., 2016). In this review, written from the perspective of an automation engineer now working in synthetic biology research and a Principal Investigator managing a research laboratory, we classify the current levels of automation in laboratories and highlight the benefits and limitations of its usage in research. We further attempt to summarise why automation has had such a limited impact in our workplace (Jessop-Fabre and Sonnenschein, 2019) and ask whether the solution to including more automation into everyday laboratory tasks may reside in greater communication between scientists and engineers. Further, we suggest that it could be accelerated by beginning with a more low-tech approach rather than striving too soon for fully autonomous systems.

## Current Laboratory Automation

**FIGURE 1:**
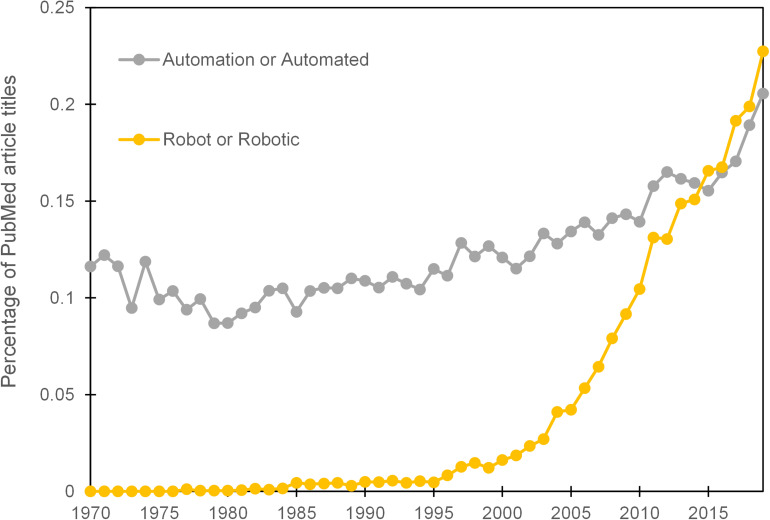
Prevalence of terms ‘automation’ or ‘automated’ and ‘robot’ or ‘robotic’ within the titles of PubMed articles per year over the period 1970–2019.

Well-meaning predictions of the cybernetic laboratory (Beugelsdijk, 1991) and a robotic revolution (Boyd, 2002) have, at the time of writing, yet to materialise in the majority of life science research laboratories. Evidence from the proportional use of the terms ‘automation’ or ‘automated’ in the titles of PubMed listed articles does, however, exhibit a steady increase over the previous 4 decades. The terms ‘robot’ or ‘robotic’, which are often used interchangeably with automation, received negligible use until the mid 90’s and then showed a more marked elevation (Figure 1). It should be noted however that, ‘robot’ or ‘robotic’ can also be used as an adjective for biological systems or medical devices and the increase in their prevalence may represent changes in language usage rather than an indication of greater automation usage. A more thorough text mining exercise than ours attempted to measure the extent of manual protocols that could potentially be automated through analysis of methods sections in published life science articles. The study concluded that 89% of articles featured a manual protocol that has an automated alternative (Groth and Cox, 2017). Whilst there is a scale of automation, from the simple to the complex, that could be applied to these protocols, such data provides evidence that there remains a large potential for automation in most biology research laboratories. There are also clear claims in the literature that researchers working in academic institutions have been slow to embrace automation (Sadowski et al., 2016; De Almeida and Ferreira, 2017; Jessop-Fabre and Sonnenschein, 2019).

In this review we focus on automation where it describes equipment that physically manipulates items and we do not consider solely software-based technologies, such as image analysis and data mining tools. Within our scope there resides a diverse range of equipment that is found in research laboratories, from simple hand tools to entirely autonomous systems. A classification system for laboratory automation equipment has, to our knowledge, yet to be published, although a number of equivalent methods have been developed for classifying industrial automation. Frohm et al. (2008) reviewed these systems before proposing their own 7 levels of automation. These levels and descriptions are displayed in Table 1, alongside examples typically seen in an academic research laboratory, and an indicative cost.

**TABLE 1:** Automation levels (Frohm et al., 2008) with example laboratory automation equipment and an indicative cost range.

Automation level	Description	Biology research lab example	Indicative cost
1	Totally manual – Totally manual work, no tools are used, only the users own muscle power. E.g., the users own muscle power	Glass washing	£0
2	Static hand tool – Manual work with support of static tool. E.g., screwdriver	Dissection scalpel	£10 – 30
3	Flexible hand tool – Manual work with support of flexible tool. E.g., adjustable spanner	Pipette	£100 – 200
4	Automated hand tool – Manual work with support of automated tool. E.g., hydraulic bolt driver	Stripette and handheld dispenser.	£200 – 300
5	Static machine/workstation – Automatic work by machine that is designed for a specific task. E.g., lathe	Centrifuge, PCR thermal cycler, spectrophotometer, gel documentation system	£500 – 60000
6	Flexible machine/workstation – Automatic work by machine that can be reconfigured for different tasks. E.g., CNC-machine	Motorised stage microscope	£70000 – 120000
7	Totally automatic – Totally automatic work, the machine solve all deviations or problems that occur by itself. E.g., autonomous systems	Automated cell culture system, bespoke laboratory equipment e.g., Labman formulation engine.	£100,000 – 1,000,000

It is noticeable from Table 1 that the majority of equipment items that researchers would consider as the most expensive in their laboratory are categorised at level 5. Higher grade 6 and 7 items are a rarity in a biological research laboratory. Whilst mid-range level 5 automation items undoubtably increase the efficiency of laboratory research, they are designed for specific subtasks in a range of protocols. These items also generally require a large amount of manual manipulation both before and after machine usage. Within the research laboratory this category of equipment is commonplace and dominates equipment budgets. A further observation can be made in that the majority of research equipment in this category performs tasks that human operators would otherwise be incapable of carrying out themselves (McClymont and Freemont, 2017). The rotation of samples at high speeds and observing microscale environments are examples of tasks that would be impossible without the use of centrifugation and microscopy equipment. Automation equipment which replaces manual handling tasks is rarer, and it the prevalence of these items where academic bioresearch facilities differ to industrial environments and clinical laboratories.

Access to high level 7 automated equipment can usually only be obtained through a pooled resource shared between across the parent organisation or wider research community; these are often referred to as biofoundries (Chambers et al., 2016; Chao et al., 2017; Kitney et al., 2019). A new automation variant of the commercial contract research organisation has also arisen recently, the cloud lab. These provide researchers with remote access to heavily automated protocols available as a pay-per-experiment service (Hayden, 2014). Cloud lab executives have made grand predictions regarding the impact these facilities will have on the future of biological research (Miles and Lee, 2018; Segal, 2019), although doubts remain regarding experimental flexibility and the resulting inhibitory effect on experimental innovation (Hayden, 2014).

## Benefits of Laboratory Automation

### Reproducibility

**FIGURE 2:**
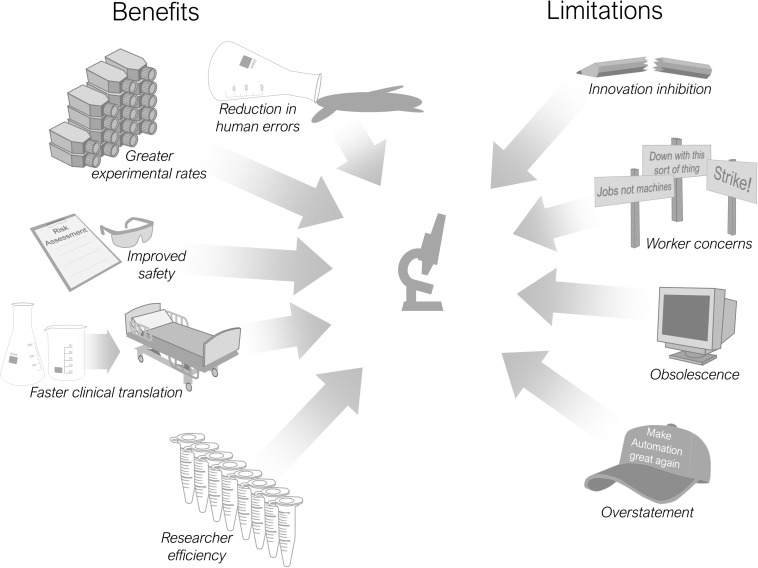
Benefits and limitations of research laboratory automation.

There are multiple advantages and limitations in including automation into scientific processes and these are summarised in Figure 2. Most pertinent is its use in improving the reproducibility of laboratory research (Kitney et al., 2019). Reproducibility is a major concern for the research community both now (Begley and Ioannidis, 2015; Baker, 2016) and historically (reviewed by Fanelli, 2018), with associated economic implications (Freedman et al., 2015) and an undermining of public trust in science (Saltelli and Funtowicz, 2017). Debate continues regarding the definition and scope of the reproducibility issue (Casadevall and Fang, 2010; Goodman et al., 2018), alongside proposed improvements in scientific practices (Peng, 2015; Munafò et al., 2017) and remedial technologies (Benchoufi and Ravaud, 2017). Increasing the use of automation throughout research laboratories is one such proposition (Jessop-Fabre and Sonnenschein, 2019; Kitney et al., 2019). An improvement in reproducibility is cited as a beneficial effect of automation implementation within clinical laboratories (Hawker et al., 2018; Genzen et al., 2018).

Automation can assist in improving reproducibility in three ways: a reduction in human-induced variability, an increase in the rate of data generation, and a decrease in contamination. The contribution each of these factors has on increasing reproducibility depends on the individual protocol. Firstly, experimental variability caused by humans is an omnipresent day-to-day reality in research laboratories (Plebani, 2010; Price et al., 2015). Variation in protocols can arise from the same person unknowingly performing a task differently each time or between different individuals attempting to carry out the same procedure. Variability that is noticed at the time can be corrected for with repeated protocols or experimental redesign, although with an associated time penalty. However, variation that goes unnoticed will manifest itself in final datasets and published results. Automation can replace many, but not all, of these human-based sources of variability. Mechanised componentry is more suited to repetitive tasks (Moutsatsou et al., 2019) in comparison to humans who are vulnerable to progressive mental fatigue (Xu et al., 2018), physical weariness (Björklund et al., 2000; Iridiastadi and Nussbaum, 2006) and also distracting influences (Varao-Sousa et al., 2018). Laboratory protocols where manual operations have been automated demonstrate greater consistency in their results, improving experimental reproducibility (Klevebring et al., 2009; Price et al., 2015). Secondly, a greater rate of experimental data capture, with an increased volume of results, can be achieved with automation alongside a wider range of experimental variables tested, including controls. Ultimately this increases the likelihood that others will be able to reproduce and build on their findings (Maleki et al., 2019). Finally, there are those laboratory protocols that are susceptible to contamination that can arise from either from the researchers themselves (Salter et al., 2014) or through increased exposure to environmental contaminants due to ponderous manual handling operations (Greub et al., 2016). Automation can remove contact with human operators (Wilke et al., 1995) or reduce potential contaminant exposure by lowering the required number of manual handling steps (Mifflin et al., 2000; Moutsatsou et al., 2019).

### Laboratory Efficiency

Efficiency is considered of paramount importance within manufacturing and can be defined as the rate of production, divided by the resources such as labour, input materials needed to accomplish this rate. By investing in automation, a company can increase the rate of production and also reduce the resources needed to achieve this rate. With a market available this can translate to a corresponding increase in profits (Ceroni, 2009). A research laboratory investing in automation can improve the efficiency of its researchers (Hawker and Schlank, 2000; Schneider, 2018) with machinery able to achieve a greater rate of experimental output than a manual based alternative (Tacker et al., 2014; Price et al., 2015; Choi et al., 2018). It should be noted that an automated protocol need not take less time from start-to-finish to result in higher output than the manual alternative, as long as it demands less human intervention (Reed et al., 2018). This is due the to the reward for academia differing from industry, with efficiency considered more as a time input to experimental output ratio. The key benefit derived from laboratory automation driven processes is therefore in the time saved by the researchers; time that can be spent on other parallel experiments. Automation in most cases will induce a transition from manual to cognitive labour (Kaber et al., 2009). Allowing an operator to set a protocol in operation and walk away to think and focus on other tasks is a valuable function for any automation equipment. Researchers frequently have multiple projects, and experimental protocols operating in parallel as well as an array of responsibilities beyond the laboratory. With a greater rate of automation-driven experimental output researchers can also identify which aspects of their experiments don’t work and adjust more quickly (Baranczak et al., 2017). Within industrial pharmaceutical development this methodology is known as fail fast, fail often (Clark and Pickett, 2000; Khanna et al., 2016; Besteman and Bont, 2019). Efficiency gains can also extend to the use of expensive reagents and materials. Automation can provide a higher level of precision in reagent dispensing, reducing the amount needed per experiment.

### Faster Translation

Automation has an important role in those laboratories engaged in applied research who are seeking to develop novel therapeutic interventions such as cell-based therapies, pharmaceutical developments or tissue-engineered constructs for implantation. Transition of these technologies from a purely research domain to final usage in a clinical setting is frequently difficult (Ochs et al., 2017; Hua et al., 2018), often referred to as translation from the bench to the bedside (Goldblatt and Lee, 2010). By considering and including automation at an early stage in the research process, crucial elements of the process can be mechanised, increasing product quality and production rates in the laboratory before the jump to manufacturing. The technological leap from laboratory-scale production to higher-volume manufacturing is therefore shortened. Researchers who include automation technologies at an early stage are subsequently better placed to upscale their processes allowing faster commercialisation rates and deployment to the clinic (Kotin, 2011; Heathman et al., 2015; Rafiq and Thomas, 2016).

### Safety

A number of protocols carried out in the research laboratory require the handling of dangerous reagents and occasionally of hazardous tooling. The manual manipulation of hazardous items places a burden on laboratories, particularly when contending with a continual turnover of short-term contract staff and students who require safety training and supervision. By assigning dangerous handling tasks to automated machinery, the exposure of humans to hazardous substances can be reduced (Movsisyan et al., 2016; Caragher et al., 2017).

### Examples of Automation Benefits

Evidence of automation benefits can be observed in recent success stories. In projects where high-throughput, reproducible results are demanded over short time frames automation has significant advantages over manual procedures. Recently a highly automated biofoundary, normally with a focus on research applications, was repurposed towards the development of SARS-CoV-2 assays for clinical diagnostics (Crone et al., 2020). Automated liquid handling equipment was able to perform an extensive array of experimental procedures at a rate in excess of those that a manual based laboratory could carry out. Furthermore, in these time-pressured experiments, automation has an advantage over manual operators who are prone to fatigue and errors, with an associated negative effect on accuracy and reproducibility. Such work also clearly demonstrates the positive impact automation can have on public health challenges. It also an example of considerate design leading to systems that are flexible enough to be rapidly adapted to meet new experimental needs. This design feature is appropriately termed ‘facility agility.’

The use of automation to improve research efficiency is also demonstrated with a system comprising a mobile robotic platform that can autonomously navigate a laboratory performing reagent-dispensing and handling operations at a range of experimental benchtop stations (Burger et al., 2020). In combination with an artificial intelligence search algorithm, the system was able to use initial data to decide on reagent combinations most likely to include an optimal reaction mix. The capacity of the robotic equipment to operate at all hours, with pausing only to charge batteries, allowed it to test five experimental hypotheses in a fraction of the time a manual research team would have required. Although it was used to answer a research question within a chemistry context the concept would be readily applicable to life science experimental laboratories. The system shares similar liquid and solid reagent handling operations to a life science laboratory as well as the common challenge of there being too many variables for researchers to explore manually in a reasonable time. A further crucial advantage of this arrangement resides in the possibility, with appropriate safety controls, of operating as a hybrid manual-automated laboratory. A staffed day shift performing high-skilled tasks requiring on-the-spot decisions could be followed by a robotic night shift carrying out the repetitive aspects of procedures.

Researchers aiming to translate stem cell-derived therapies towards clinical applications have considered automation for a range of projects. Such therapies will ultimately require the expansion of stem cells on a scale that is uneconomical for manual based laboratories, with large numbers also needed for research and clinical trials phases. The need for reliable methods of high-volume, quality-assured cells has led to the development of automated systems such as the StemCellFactory (Doulgkeroglou et al., 2020), StemCellDiscovery (Jung et al., 2018) and AUTOSTEM (Ochs et al., 2017). The objective of these systems is to automate the normally manual stages of stem cell seeding, growth, colony selection, passaging, quality assessment, harvesting and potentially in later applications differentiation. In a similar fashion to the previous mobile robotic platform example, complex control algorithms are also being applied to these systems with the aim of improving cell yields and quality (Egri et al., 2020). These projects are an important link between the domains of basic life science research, clinical application, and commercial cell product manufacturing. By developing these systems researchers have been able to generate high quantities of cells for research and testing purposes, hastening the route to clinical usage.

## Limitations of Automation

### Incorrect Application

Despite the range of benefits that laboratory automation can bring, there remains a number of limitations. Integrating automation into a research laboratory is not in itself a guarantee of success and, where applied incorrectly can even result in even less efficiency (Zielinski et al., 2014). The nature of automated tasks also allows for rapid propagation of errors. An example would be a machine incorrectly dispensing a reagent repetitively which can then, if undetected, be distributed across many thousands of samples. In addition, the incorrect application and operation of automation may not improve the reproducibility of research between laboratories. Automation machinery carrying out the same experimental protocol in different laboratories may still produce different results. This can be due to variations in input materials, different equipment models or set-up and calibration errors. Even where automation has been carefully integrated into a laboratory and has demonstrated an improvement in reproducibility an inherent machine to machine variability can remain. What is more, this variability can be more hidden than more easily observed manual procedures. Careful maintenance, calibration and quality control measures are therefore essential in implementing any laboratory automation system (Hawker and Schlank, 2000; Xie et al., 2004).

### Obsolescence

Obsolescence is an inevitability for any technology and even, it can be argued, for scientists themselves. Many facilities will feature a dusty machine in the corner that is unused, because components and materials are no-longer available, the protocol itself has been supplanted or simply newer more effective equipment has taken over (Croxatto et al., 2016). Predicting how and when a machine will become obsolete is an inherently difficult task in rapidly evolving research fields and can be specific to individual laboratories. Some researchers will find equipment is no-longer useful after a few years of operation whilst others may continue to happily use the same machine for decades. It is not only advances in hardware and software design that can render laboratory equipment obsolete. Scientific progress in reagent properties and resulting modifications to protocols can also be responsible. The advent of new thermostable polymerases obsoleted a whole generation of Polymerase Chain Reaction machinery designed upon a more repetitive protocol (Hawker et al., 2018). Despite these difficulties, with considerate design allowing for reconfiguration and modification premature obsolescence can be delayed (Harrison et al., 2007; Crombie et al., 2017), referred to in some industries as future-proofing. Understanding and planning for obsolescence is therefore an important part of any automation strategy.

### Innovation Inhibition

There is a danger that automation can inhibit creativity in the experimental design process by limiting the opportunities for changing or tinkering with a protocol. A researcher may be less inclined to alter a protocol to optimise it for a new situation where a large number of steps are automated. This can be based upon the assumption that process steps carried out by machinery are already optimised and require no further improvement. They may also feel less able to begin changing things because they lack the confidence or maybe even the authorisation to open the box and begin modifying what is probably an expensive machine. Sharing of the machine with other users for whose purposes it is already optimised is also a brake to experimentation with parameters. Innovation inhibition is also a concern where protocols are outsourced to third party automated laboratories (Hayden, 2014).

### Workforce Impact

When integrating new automation into any workplace environment, the impact on workers and how they view new machinery must be carefully considered. Beginning in the rural English midlands with the machine breaking Luddite movement (Roberts, 2017), societal resistance to automated machinery replacing manual labour and the threat it poses to livelihoods understandably continues into the present day (Jones, 2013; Autor, 2015). Both positive and negative reactions to the introduction of automation have been observed amongst long-term workers in clinical laboratory settings (Thomson and McElvania, 2019) and it is reasonable to anticipate that similar reactions may arise in research laboratories. The outright replacement of researchers by automation is unlikely as they are currently categorised as being amongst the lowest risk of being replaced (White et al., 2019), due to their breadth of skills, including planning and creativity (Reeves et al., 2019). However, researchers solely employed to perform repetitive manual tasks are more at risk and thus more likely to view automation as a threat. Those researchers with a multitude of other protocols and tasks beyond the laboratory are more likely to view automation assistance in their day to day roles in a positive manner. The short-term contracts that predominate in research will also lessen any hostility to automation. Employees who understand that they will be moving on to another position, will see a machine as more likely to be a replacement for their replacement rather than a replacement for themselves. Although the levels of militancy advocated by the early Luddites may not be repeated, laboratory managers who introduce automation will still, like their industrial and clinical counterparts, need to be sensitive to workforce reactions, particularly the impact on any long-term employees.

### Automation Hyperbole

Both vendors of automation equipment and researchers must also be wary of overstating the benefits of automation and elevating expectations regarding the impact its introduction will have on future work practices. Automation hyperbole and the accompanying benefits is however part of a wider trend that is not only restricted to research (Wajcman, 2017). Whilst automation can improve protocol reproducibility and efficiency the individual researcher will, in the majority of cases, still be responsible for correctly operating the equipment, with maintenance, quality of input materials, and calibration. These are tasks than can require a high level of personal discipline and tenacity. With notable exceptions (King et al., 2009; Williams et al., 2015), automation will also be unable to undertake the overall experimental design and analysis. Journal publications have a responsibility too, to ensure that articles advocating laboratory automation equipment also highlight the limitations of their technologies, as well as identifying author conflicts of interests (Miles and Lee, 2018). Greater awareness of limitations will allow more effective matching of automation solutions with laboratory problems and increase the trust between commercial vendors and academic institutions.

## Laboratory Automation Obstacles

### Automation Is Expensive and Difficult to Justify

The most significant hurdle for PIs wishing to integrate automation systems into their laboratories is, unsurprisingly, cost. Commercially available automation equipment is expensive, whilst bespoke equipment for individual protocols costlier still. Cell culture is an example of a common, labor-intensive protocol familiar to generations of researchers. Equipment to automate cell culture is available and can save many hours of researcher effort from the process, but is tantalisingly out of reach for most laboratories. The cost of these items can be in excess of $1 M for a complete process system (Storrs, 2013) placing them far beyond the reach of the majority of academic laboratories. Despite being commercially available for over 18 years (Kempner and Felder, 2002) they remain a rare sight in research environments but are used in high volume cell-banking organisations (Wrigley et al., 2014; Archibald et al., 2016; Daniszewski et al., 2018).

The development of automation equipment can be a time-consuming and expensive process. Initial rounds of iterative conceptual and prototype design and testing are followed by final design, build, and commissioning phases. Coordination is needed from a variety of disciplines including mechanical, electrical and software engineers alongside close collaboration with the end user. Most important for all automation projects however, is a source of capital investment. Industrial investment in automation is matched to business cases in which increasing confidence in the product and the associated income from projected sales is used to justify upfront capital expenditure. However, an academic principal investigator seeking to invest in automation for their laboratory is confronted by a different set of challenges. When compared to industrial and commercial organisations, a research laboratory’s output or success rate cannot be measured in using the same readily quantifiable metric of profit. Indeed, academic research output has long been a difficult entity to define both for individual researchers (Klaus and del Alamo, 2018) and laboratories (Kreiman and Maunsell, 2011; Abramo and D’Angelo, 2014). It is therefore more difficult to construct a ‘business’ case when seeking funding for laboratory automation equipment. A factory manager is able to justify a new item of automation based upon the argument that whilst it may initially cost *X* units of currency it will increase profits by *X* + *Y* units, measured in the same currency (Ceroni, 2009). A clinical laboratory manager can present a similar case based upon both cost (Archetti et al., 2017; Sarkozi et al., 2003) and the quantifiable output of turnaround time (Hawkins, 2007; Archetti et al., 2017). A research laboratory manager however, in the same position applying for funding, will have greater difficulty in arguing that although the proposed equipment will cost *X* units of currency it will increase their laboratory’s research output by *Y* vaguely defined research outputs. The ambiguity of research success hinders laboratories seeking to invest in automation.

### Research Funding Structures

The allocation of scientific funding to academic institutions further limits investment in automation. Research programs are most frequently funded through externally sourced grants that are applied for in a competitive environment, with pre-applied constraints on the amounts available and where these funds may be spent. Understandably the majority of funding calls open to scientific laboratories are seeking answers to novel scientific questions and not looking to develop items of equipment that are essentially engineering challenges. Should an applicant wish to include standard or bespoke automation when applying for grants, capital expenditure on large equipment, if even permitted, must be explicitly accounted for before the project starts. Unfortunately, the nature of research means that the details of protocols needed for the project are not always available during the early proposal phase. Estimating the both the timescales and cost of automation at such an early stage is a difficult task for supervisors of biological research laboratories who will have limited experience of budgeting for automation hardware. The time duration of funding grants also limits the development of automation, usually with the maximum being 5 years (European Commission, 2016; Vaesen and Katzav, 2017). Automation strategies for industry are generally greater in duration and aligned to the anticipated lifecycle of the product, frequently extending into decades. In the case of commercialising a novel pharmaceutical product or medical device the automation strategy can be aligned to the 20-year exclusivity patent window. Automation expertise acquired over this time can then be exploited to maintain a competitive advantage when the window expires. Academic projects of a comparable length are rare. The Human Genome Project is one exception, and consequently was able to invest and substantially benefit from automation (Meldrum, 2000). However, long-term, project specific funding stability is rarely available to most academic principal investigators, limiting automation investment.

Short-term research funding also places a limit on the individual researcher’s ability to develop automation. Hands-on researchers are best placed to determine which elements of their protocols would benefit from automation. However, these individuals are typically Ph.D. students or early career researchers with a time-limited contract or project. Such temporal limitation leaves little room for developing an idea for protocol automation into a functional system, particularly with specific scientific targets attached to the grant scheme funding their project. Short duration research positions reduce not only the time available to develop novel automated laboratory equipment but also the motivation for doing so. On completion, a researcher is likely to move on to a new laboratory contract or a career beyond academia (van der Weijden et al., 2016). Researchers are therefore unlikely to experience any of the long-term benefits from planning automation. The cumulative effect of short-term, competitive grant allocations and transient researchers creates an environment unsuited to the long-term financial investment required for laboratory automation development.

A limited number of large grant funded projects have been successful in devising automation strategies and equipment, although often with a focus on industrial scale systems for clinical translation rather than research laboratories. One area that seen recent attention is the aforementioned development of high-volume manufacturing solutions for the production of Mesenchymal and Induced Pluripotent Stem Cells to meet anticipated future clinical demand (Marx et al., 2013; Panchalingam et al., 2015; Rafiq et al., 2016; Ochs et al., 2017; Jossen et al., 2018). It is hoped that technology developed in these programs will, in the future, trickle down into more affordable systems that can be exploited by smaller research laboratories.

### Stifled Commercial Development of New Laboratory Automation

Financial challenges also hinder those commercial organisations seeking to develop laboratory automation equipment. Industrial automation design and development is often a bespoke, collaborative arrangement for a particular challenge. A manufacturer will approach one or more automation developers to design a manufacturing system for their product. In this scenario the manufacturer is usually a much larger organisation with abundant reserves of capital and will also carry the majority of the risk should the product not sell as well as expected. To aid in mitigating this risk they are able to utilise their marketing, sales and distribution expertise within their particular market sector. For development of automated laboratory equipment, the scenario is often different. An automation developer may wish to partner with an academic research laboratory. However, as previously detailed, in such an arrangement the laboratory will be unable to operate as a cash-rich development partner unless a substantial funding grant can be obtained. The automation developer must therefore carry the risk that the equipment will not be commercially successful and assume the role of marketing and selling the product to the wider research community. Biological laboratories are best placed to identify where certain processes would benefit from automation, but don’t have the financial resources or expertise to develop these systems themselves. Automation companies, whilst having the capable expertise to develop automation equipment will be reluctant to pursue such a business strategy requiring up-front investment to develop a product for customers widely acknowledged to have little disposable capital.

Small-to medium-sized automation companies have often been most successful at innovative development of laboratory equipment, funded through grant schemes in cooperation with an academic institution or external venture capital funding. Examples include benchtop pipetting systems from Andrew Alliance and OpenTrons and Labman automation’s formulation engine. Access to joint research grants and funding schemes can encourage the development of novel automation solutions by increasing industrial and academic collaboration whilst also reducing the risk the commercial risk that developers are exposed to.

### Laboratory Space

Alongside the financial investment required for automation researchers must also find physical laboratory space for new equipment, incurring a footprint cost (Wong et al., 2018; Moutsatsou et al., 2019). The size and mass of many automation items means that it is not always practical or safe to tidy the item away and store it when it is not required. Laboratory space is often at a premium in many research institutions with territorial researchers often coming into conflict over the allocation of it (Adams, 2004). A bench occupied by equipment is also an area that could be otherwise be utilised by productive researchers. The requirement for some laboratories to operate as a dual research and teaching environment further constrains the available space. It may also not be possible for automation to totally replace more manual based equipment and space in laboratories, with room required for both. The need to maintain cell culture hoods for teaching is one example. Developers of laboratory of automation have attempted to minimise the footprint of their machinery through innovative reworkings of traditional laboratory procedures. The use of hollow fibre arrays (Russell et al., 2018) and multi-axis liquid and labware manipulation (Kato et al., 2010) are examples of compact automated adherent cell culture systems. Spatial constraints may push future bench-based laboratory automation towards an architectural style resembling inner city skyscrapers.

### Protocol Variation and Usage

The very nature of bioresearch involves the design and implementation of protocols aimed at the determining answers to novel research questions. In pursuit of these targets, researchers will devise new protocols or substantially modify existing ones to suit their needs. Recurring cycles of method generation and evolution within the research laboratory create a high-level of protocol variation that is not always easily automated. Matching commercially available automation equipment to these requirements is often not a feasible option with fixed componentry and locked-in software frequently being the limiting factors. Automated cell culture is an example where the available systems can be insufficiently flexible to accommodate the specific cell culture requirements of an individual laboratory (Crombie et al., 2017), with some requiring a broad range of cell culture types and others having more focussed needs. A high level of experimental process variation is therefore more likely to require a bespoke automation system, the development of which will have an associated time and financial cost. Clinical laboratories, by comparison, have a greater level of consistency across protocols both within individual laboratories and across institutions, contributing to the widespread implementation of automated systems. High process variability is also cited as one of the major challenges for integrating automation into existing industrial environments (Frohm et al., 2006) and is necessary when adapting to changing market conditions (Froschauer et al., 2008). Across laboratory protocols there are process steps that are common, and it these where commercially available systems are more likely to be of assistance to the individual researcher. Liquid handling, through the manipulation of pipettes and receptacles is a one example ubiquitous to a range of molecular biology protocols, with a growing number of competing vendors offering more affordable and adaptable automation options (Barthels et al., 2020).

How frequently a protocol is likely to be used over time is also a key factor when considering automation. A protocol developed for a specific project may only be used in a single laboratory for a short period, negating the long-term benefits that automation could provide. On occasion a researcher may find that their new protocol becomes widely adopted for an extended period in their own laboratory, and possibly throughout other laboratories too. In this scenario automation becomes a more attractive option and is not always driven by the original founding laboratory. Sequencing, is one example where the initial manual protocol developed by Sanger and colleagues (Sanger et al., 1977) was eventually automated by researchers at different institutions (García-Sancho, 2007).

### Labware and Consumables

Automation equipment operates most effectively when input materials or consumables are standardised. In the case of standard shaped labware this allows non-adaptive, rigid automation components such as grippers to gain full custody of the device, allowing greater accuracy of placement and potentially faster actuations. Currently there remains a large amount of variation in labware not only between research laboratories but also within the same laboratory. The variant a researcher uses can change frequently based upon cost, availability or personal preference. Disposable plastics are an example where different manufacturers produce products that are, from an experimental, viewpoint functionally identical but with variations in the products dimensions and materials. The justification for these variants maybe a small improvement in handling, or simply to circumvent intellectual property assigned to a competing product. These present a significant challenge to automated handling equipment where even small variations, that are unnoticeable when handled manually, can render an automated system using non-adaptive handling elements useless. Clinical laboratories negate this issue by utilising standardised plastics for sample collections that can then be more readily processed autonomously. The recent advent of soft robotics may provide solutions to these challenges where rigid handling systems are replaced with pliable, adaptive designs sometimes based upon biomimetic examples (Noel and Hu, 2018).

A counterstrategy to labware variation has emerged from commercial developers of automation. Unfortunately, the solution is often combined with a sales strategy aimed at securing a continuous revenue stream following the sale of the initial capital equipment. Commercially available systems are frequently designed in a fashion such that automation systems can only operate with specific consumables, available for purchase from themselves or a licensed distributor (Huggett et al., 2009; Moutsatsou et al., 2019). Examples include the pipette tips for the Opentrons and Tecan EVO liquid dispensing systems, array tape for Douglas Scientific’s IntelliQube PCR system, purification cards for Invitrogens benchpro and spin kits for Qiagens Qiacube system. A laboratory binding themselves to a single consumable supplier has little or no guarantee of future price stability or even long-term supply should the commercial vendor cease to exist. Committing to a long-term, single vendor, supply chain is considered a very unwise strategy in a commercial context but is a worryingly frequent arrangement for automation equipment available to research laboratories.

**FIGURE 3:**
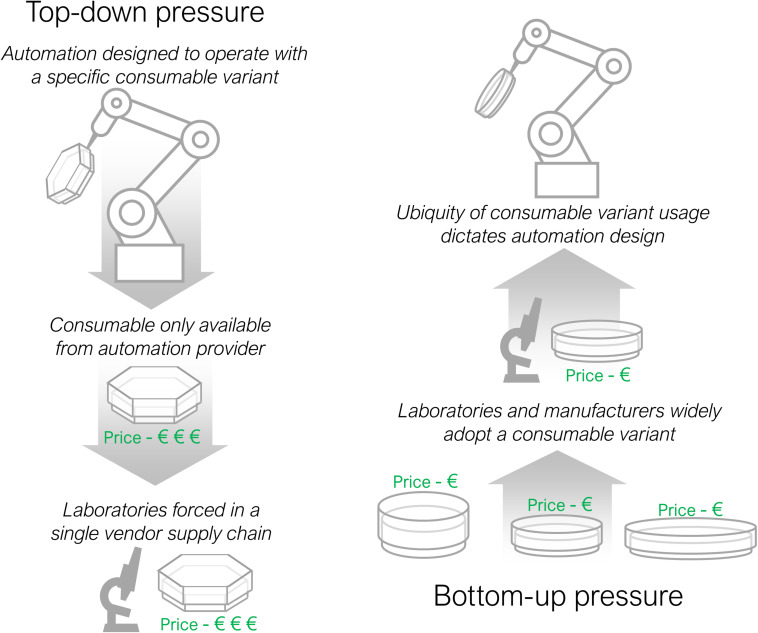
Top-down and bottom-up consumable adoption pressures. Top-down pressure occurs when an automation developer imposes a consumable on laboratories through tooling specific design. Bottom-up pressure acts in the reverse direction with laboratories and automation suppliers coalescing behind one consumable variant that then determines the design of automation equipment.

There are two competing forces for labware standardisation; top-down and bottom up pressure, outlined in Figure 3. Top-down pressure, as described above, is where commercial automation organisations seek to dominate a section of the market by forcing users to purchase specific labware through the sale of inflexible hardware. Bottom-up pressure acts in the opposite direction, when manufacturers of labware and laboratories slowly gravitate towards one standard form that automation developers are then forced to adopt. An example where bottom-up pressure has succeeded is in the largely standardised external dimensions of well plates, the ANSI/SLAS standard (Society for Laboratory Automation and Screening, 2011), that has enabled automation of microscopy and plate reading procedures (McClymont and Freemont, 2017). The range of automation equipment available for standard well plates is correspondingly larger, increasing competition, reducing running costs and making automation more affordable. There is likely to be a reciprocal benefit for labware manufacturers too, with an associated increase in demand for consumables. More instances of labware standardisation would allow a wider range of protocols to be automated.

### Environment Impact

The environmental impact that an item of equipment can have throughout its entire lifespan, from manufacture, to usage, to end-of-life disposal and recycling is an important consideration for many research institutions. A particular concern for laboratories is the rate at which automation consumes disposable plastics. Research institutions produce a large amount of plastic waste, estimated at 5.5 million tonnes annually (Urbina et al., 2015), primarily to avoid contamination between samples. Commitments to minimising their use are part of a growing trend where laboratories aim to switch to recyclable or reusable alternatives (Bistulfi, 2013; Krause et al., 2020). Automation designed around the same single-use plastic principle can generate even greater volumes of waste than human operators, due to higher experimental throughputs (Howes, 2019). These designs are incompatible with research organisations who are committed to minimising their environmental impact. The consideration given to environmental concerns is currently very low or non-existent in many commercially available laboratory automation systems. An exception is Grenova’s pipette washing systems (Safavi and Anderson, 2019) that can be integrated into existing automated liquid dispensing units. It is hoped that this type of equipment represents an emerging category of environmentally focused automation that will become ever more important to laboratories in the future.

### Culture

There exists a fundamental culture difference between an academic research laboratory and the industrial workplace environment, that can inhibit investment in automation. It is hoped that the majority of principal investigators view their laboratory as a platform for staff and students to increase their skills and experience before they move onwards in their careers. This is a crucial ‘people’ output that accompanies the research output of a laboratory usually measured in scientific discoveries and publications. Although many companies also place a high-value on workforce upskilling their focus is primarily on profit and not on being a training institution to allow employee progression elsewhere. Consequently, many will favour investment in equipment over staff if a business case can be made (Rampell, 2011). An academic principal investigator however, is likely to preferentially invest in additional people rather than equipment, with funding schemes frequently weighted this way too. Money spent on a large item of automation equipment could, for example, pay for several post-doctoral researchers or fund multiple Ph.D. projects. In the context of automation this culture could be described as a form of benevolent Luddism.

The availability and culture of undergraduate labour may also be inhibiting investment in laboratory automation. Undergraduates working in laboratories contribute by performing experiments that can generate preliminary data for grant applications or for publications. The benefits to the student reside in the acquisition of experience and skills that can enhance their employability prospects upon completion of their studies (Seeling and Choudhary, 2016). This reciprocal arrangement and the high availability of undergraduates provides a means for carrying out labour intensive laboratory tasks. Not all principal investigators will view this relationship in such a cold manner, and will considerately assign duties that can generate useful data whilst simultaneously teaching students both the basics and realities of research. Unfortunately, there is evidence that some less altruistic supervisors do assign undergraduates to tasks that require a high degree of repetition (Hayward et al., 2017). These are likely to be precisely the type of tasks where automation can be effectively applied.

## The Laboratory Automation Interim Technology Gap

**FIGURE 4:**
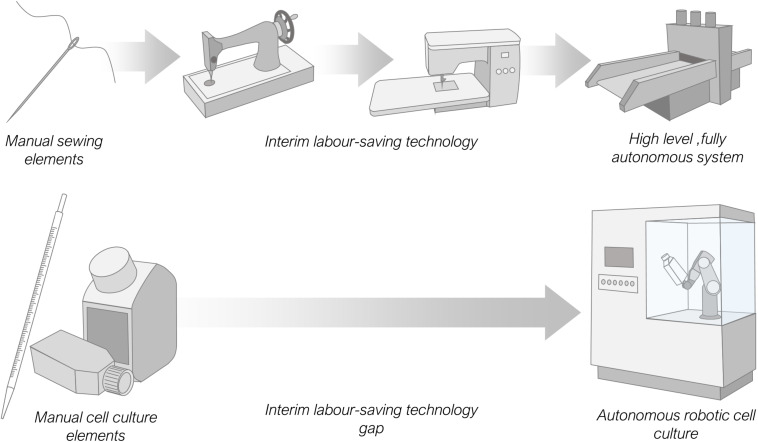
Comparison of available labour-saving automation options for the manual intensive processes of sewing and cell culture. Sewing has a range of interim automation options up to fully autonomous systems. Cell culture by contrast has only high-level automation equipment and no interim low-cost analogues to replace or augment manual labour.

It is interesting to compare the relatively recent development of manual labour-saving laboratory automation equipment with other older, more mature automation processes. Here we refer to equipment that replaces manual human manipulation rather than machinery that performs operations operators are physically incapable of executing, such as centrifuging. Taking the millennia-old example of sewing, with just a needle, thread and cloth it is possible, given time, for a skilled human operator to create a garment. Equally the same items can be completely mechanised with expensive, high-level automation equipment and the garment produced with no human input necessary beyond the need to turn the machine on. Comparing with the laboratory process of cell culture which requires, media, pipettes, labware and some starting cells a skilled operator can also, given time, passage cells and create a sub-culture for experimentation. Again, the same output can also be produced using an entirely automated, costly, high-level system, with minimal operator input. However, in the case of needlework there exists a range of lower cost interim labour-saving automation options between these two extremes, such as motor driven stitching machinery, or manually powered mechanisms, exemplified in the Singer sewing machine (McLoughlin and Mitchell, 2013). This is not currently the case for cell culture, there are no examples of commercially available low-cost machinery (Figure 4).

Interim automation can arise in several scenarios. More commonly it occurs incrementally over time, as technological advances permit a shift from simple to complex machinery. Alternatively, on occasion a high-end complex automation system may be simplified due to new demands, such as an economic demand for cheaper equipment. For many laboratory automation processes there has been a rapid leap from simple to complex with, as yet, little or no development of lower cost automation technology. We believe this is due in part to the reasonable desire for academic laboratories and companies to be seen to be developing equipment at the forefront of technology. In simple terms, low-cost interim automation that removes some but not all of the manual labour from a protocol is not fashionable enough. It is unlikely to lead to a prestigious journal publication and, for commercial organisations, will not lead to financial rewards, with likely low sales volumes and low profit margins. There are therefore few incentives for academic and commercial automation developers to design such equipment.

## In-House Laboratory Automation

Despite the hurdles facing researchers wishing to automate elements of their experimental procedures, there are many examples where laboratory automation development is carried out ‘in-house,’ without the assistance of a commercial partner or a large automation dedicated funding grant. Research teams are recognising that their protocols could be made more efficient by including automation but find themselves restricted financially and functionally by commercially available options (Pilizota and Yang, 2018). A range of ingenious methods have been developed to build low-cost automation solutions, including the integration of Lego into microscopy automation (Almada et al., 2019), microfluidics for DNA assembly (Shih et al., 2015) and rapid synthesis and testing of small molecule libraries (Baranczak et al., 2017). Laboratories with novel protocols that are nearly but not quite suited to existing automation equipment have been able to successfully upgrade commercially available systems for their specific needs (McGraw et al., 2014; Richter et al., 2015; Zhang et al., 2016; Crombie et al., 2017; Konczal and Gray, 2017). Repurposing existing equipment in this fashion either through software or hardware modification is a cost-and time-efficient method of obtaining higher levels of protocol automation without the arduous task of designing and building an entirely novel system. The number of automation development tools, components and virtual training options available to research laboratories continues to broaden, increasing their capability to develop low-cost solutions to labour intensive processes. The advent of affordable 3D printing modalities (Jones et al., 2011; Zluhan et al., 2016; Capel et al., 2018), off the shelf actuators and readily programable microcontrollers (Mabbott, 2014; Kim et al., 2015; Wong et al., 2018) has given research laboratories the ability to produce componentry that can then be assembled, controlled and automated all for a relatively low cost (Courtemanche et al., 2018; Needs et al., 2019; Barthels et al., 2020). Open source designs and software have an important enabling effect for researchers who may not have engineering or programming expertise. Researchers are also able to exploit the growing market for second hand laboratory automation equipment (Zluhan et al., 2016), a case of one lab’s trash is another labs treasure. Developing automation internally, whilst often cheaper, and potentially a more rewarding and enjoyable process (Pilizota and Yang, 2018) can however require a substantial investment in time (May, 2019). That laboratories are frequently forced into developing their own systems is an indication of the paucity of commercially available options. Existing automation developers see an insufficient market for providing their services and expertise to develop bespoke items for individual laboratories and will be justifiably reluctant to provide open source solutions that may compromise their intellectual property.

## Remedies

Increasing the quantity and quality of laboratory automation within the research laboratory will require a concerted effort from funders, research institutions, automation developers and researchers themselves. The desire to automate elements of laboratory protocols exists. Researchers and their governmental funders (Reeves et al., 2019) collectively recognise that mechanisation can improve reproducibility and efficiency. When attempting to develop laboratory automation three interrelated components are needed for success. Connecting researchers with automation needs to automation engineers, financing the resulting collaboration, and ensuring the resulting design meets the needs.

### Collaboration

Encouraging academic researchers to engage and collaborate with industrial organisations has been a long-standing objective for their host institutions. Such joint enterprises are hindered by the significant differences in culture and attitudes to one another (Berman, 2008) which are in part due to each partner having different timescales and expectations from projects. Academics build projects slowly through the funding stages and ultimately desire experimental data that can be packaged into publications. Industry often likes to move more quickly and would like intellectual property that can be reconstituted into a commercial opportunity (Lynch, 2016). Contrary to widespread belief these viewpoints are, however, not always the most prominent motivations for collaboration, with altruistic aims also prevalent in both parties (Berman, 2008).

Automation engineers and life science researchers operate in markedly different disciplines and in different work environments, rarely occupying the same space to share problems and ideas. Events where these disparate groups can be brought together would allow new ideas and projects to develop, in a similar fashion to academic conferences encouraging collaboration between different laboratories. Automation engagement events that feature all levels of employees from both sides of the divide would have the greatest effect. Interaction between industrial managers and academic supervisors as well as researchers who are researching and engineers who are engineering could allow the development of solutions to everyday automation challenges in the laboratory.

Collaboration can also be an internal academic arrangement. Life science laboratories often have a source of automation engineering expertise within their own institution in the form of engineering faculties. Both disciplines could benefit from increased interaction and discussion around laboratory automation, with examples of collaborating biomedicine and engineering departments producing innovative automated equipment (Kato et al., 2010; Kane et al., 2019). Collaboration at an educational level can be beneficial too. Allowing undergraduate engineering students to undertake projects based upon automating a protocol within a laboratory would provide the host laboratory with designs and automation aids. Interdepartmental, interdisciplinary collaborations can bring benefits for students too, providing real world problems to develop their skills and the opportunity to apply theoretical knowledge (Wilson and Zamberlan, 2012).

More varied career paths that allow employees with experience of industry-based automation to work in research environments can also develop new ideas that lead to mechanised laboratory equipment. Academic and industrial career paths diverge at early career stage and rarely reconnect. The majority of professional individuals progress from an academic institution into an industrial or commercial organisation. Researchers typically remain within a university environment accruing the required qualifications and experience as their career progresses. Reverse flow of employees, where an individual moves from industry to academia is less common (Bonner, 2006). Encouraging a greater level of employees with experience of automation to work within life science laboratories will promote an exchange of ideas that can lead to experimental mechanisation. Such employee exchanges need not be permanent and can be sabbatical-style placements targeted at a specific project. The Knowledge Transfer Partnership is one successful long-running academic-industry exchange scheme in the authors host country that allows an employee to concurrently work on a project at both an academic and industrial organisation (Howlett(ed.), 2010). These types of employee arrangements have a further benefit in deepening the relationships between Universities and industrial organisations. Academic institutions that can successfully foster relationships with industrial partners can reap substantial rewards not only in the form of publications and possible financial licencing agreements but greater reproducibility too (Edwards, 2016). In a notable success story, automated sequencing technology, now the mainstay of genetic research, was successfully developed at Caltech, a research organisation with strong links to industry (García-Sancho, 2007). Ultimately though any collaboration, regardless of the method of inception, is unlikely to succeed or even be embarked upon unless both partners are confident that they have the financial resources to proceed.

### Funding

Greater implementation of automation can bestow benefits to funding organisations. Devoting financial resources towards automation engineering may seem paradoxical where the long-term objectives are targeted towards developing therapeutic interventions for biological diseases. However, the reproducibility of published research is essential for research financed by these organisations. Automation is a critical component in driving upwards the reproducibility of disseminated research (Winder, 2019). In addition, as research confidence increases in a particular therapy consideration will eventually need to shift towards how the technology can be produced in sufficient quantities and at an affordable price so that it is available to the greatest range of patients. As previously discussed, including automation at earlier stage in the development process can help in attaining these goals, easing the transition from the experimentation phase to clinical usage. Competitive schemes, where funds are specifically are made available for developing laboratory automation would be beneficial in bridging the distance between the lab bench and the bedside.

Automation can provide benefits too for governments funding academic institutions. Increasing the level of automation across workplaces is acknowledged as strategy for economic progress (Velásquez et al., 2009; Reeves et al., 2019) with research laboratories being no exception. Access to higher levels of automation increases the output of research laboratories that exist in publicly funded institutions. Any associated automation dividend will also require appropriately skilled technical staff to maintain, operate and enhance laboratory equipment. A greater range of dedicated grant schemes specifically targeted at developing laboratory automation will, in the long-term, increase the effectiveness of all research funding.

### Laboratory Automation Design

Improvements can be made in automation design, how it is implemented in laboratories and the range of available automations options. A large amount of laboratory automation is based upon an anthropomorphic design framework that mimics human movement. Expensive laboratory equipment frequently features an over reliance on robotics to manipulate tooling, reagents and labware in a similar manner to how researchers would themselves. These types of designs can present as being visually high-tech and impressive and there is indeed an advantage to machinery that presents as more human-like in that it is more likely to be trusted by human operators (de Visser et al., 2016). Unfortunately for many applications these designs are not always the most efficient means for automating a laboratory protocol. Robotic actuators featuring multiple axes and large operating envelopes also require even larger guarding enclosures and correspondingly complex control systems (Yachie and Natsume, 2017). These design attributes render such equipment spatially and economically unsuitable for the majority of research laboratories. McClymont and Freemont provide an example where an assay requiring liquid handling can be more effectively processed and multiplexed with tooling that is not based upon an anthropomorphic design (McClymont and Freemont, 2017). Hollow fibre cell culture systems are further examples of automation systems that have successfully eschewed more traditional anthropomorphic designs (Eghbali et al., 2016).

Designing for flexibility is also an important factor for laboratories where there is a high level of protocol variation. Laboratory automations systems designs that anticipate future scientific developments and allow for subsequent adaptation will be less likely to become prematurely obsolete and thus more valuable to research laboratories. Machinery based upon modular based design is one approach to a flexible system. Modular automation systems can allow selective matching of automation to the protocol requirements, minimising the purchase of redundant features, and also providing the option for future upgrades should it be needed. There are indications that laboratory automation developers are becoming more aware of the need for flexibility. The ongoing development of technology such as Formulatrix’s rover system is one example where microwell plates are autonomously transferred between processing modules in a novel reworking of the robotic warehouse concept (Wikholm and Lindblom, 2019).

The capability for an automation system to be modified without specialist engineering knowledge is desirable too. Allowing researchers to automate a wider range of process steps without the need for time consuming and expensive tooling redesign or extensive software reprogramming. An interesting extension of the modular design approach is to unify existing automation equipment so that it capable of performing the desired protocol in one continuous process stream. The recent development of software by the company Synthace that is capable of communicating and linking robotics from different manufacturers is one promising system for laboratories requiring highly flexible systems (Sadowski et al., 2016; Jessop-Fabre and Sonnenschein, 2019).

To reduce the manual labour burden on laboratory research staff and students there is a need for a broader range of automation equipment. These designs should target the identified gap in labour saving automation with a focus on reducing price and footprint. In this regard employing multi axis robotics may not be the most optimal design solution and developers should be prepared to explore more cost-effective, low-tech routes to protocol automation, even if seems like a less fashionable option.

## The Future of Laboratory Automation

It is with a certain degree of trepidation that we follow in the footsteps of others and attempt to predict the future of laboratory automation. The life science research laboratory of the future will undoubtably feature more automation equipment. How quickly automation is adopted will in all probability be slower than many would like and haphazard, with some fields being more suitable than others. Many of the obstacles to laboratory automation ingress we have described are long-standing and hardwired into the working practices of academic research. In particular financial hurdles faced by individual principal investigators are unlikely to be resolved and overcome in the immediate future. Bespoke, high-level automation solutions will remain beyond the reach of all but the most monied laboratories for a considerable time. Greater progress can be anticipated in the design and price of lower-level automation equipment. It is reasonable to assume that like other technologies laboratory automation will continue to mature with falling prices and more user centred designs. Hopefully incorporating more flexibility in response to consumer demand. In part this progression is already underway, with promising releases of low-cost liquid handling platforms and ongoing development of modular systems. The demand from research laboratories for automation that seeks to limit its impact on the environment will grow considerably and it is hoped that developers will create and adapt their designs to meet this need. Life science researchers will also continue to develop their own homemade laboratory automation and repurpose existing equipment, encouraging other laboratories to also take the leap into engineering. We predict that the second hand market will become an important resource for those choosing this route to automation.

Access to pooled resource, high-level, automation in the form of academic biofoundries is increasing and will continue to do so with expansion of existing facilities and the foundation of new ones. The outsourcing of protocols to commercial cloud laboratories has been predicted to become commonplace for a huge range of life science laboratories. From the perspective of the lab bench we are more circumspect in regards to the impact these organisations will have on day to day experimental research, with experimental range and flexibility key issues. Ultimately, the marketplace laws of supply and demand will dictate the success rate of these enterprises.

An appreciation of the limitations of automation both generally and for items of specific equipment is needed from academic, commercial and funding organisations and individuals. Of all the limitations discussed in this review we wish to particularly highlight the danger of innovation inhibition. Innovation in the laboratory is essential and the freedom to tinker and create new protocols needs to be retained if research is to retain a high degree of novelty. Ensuring that automation remains compatible with the curiously minded researcher will be a significant challenge for our field in the future.

In response to automation ingress the skills of life science researchers will need to adapt. The presence of more automation equipment will require more engineering type-skills to ensure correct equipment operation and implementation of protocols, along with a working knowledge of the biology under experimentation. Researchers will therefore need both biology ‘wet’ skills and ‘dry’ automation skills; such people have been imaginatively titled amphibious researchers by Mellingwood (2018). It is therefore likely that automation will spawn a new generation of researchers with a range of interdisciplinary skills.

In summary, automation in life science laboratories lags behind its industrial and clinical counterparts due to an array of inhibiting factors, including financial, spatial and cultural challenges. Those who are able to surmount these barriers and integrate automation into their everyday protocols can reap significant reproducibility and efficiency benefits. It is essential that future laboratory automation systems are designed for flexibility to permit adaptation for changing laboratory needs and prevent the stifling of protocol innovation. A wider range of affordable bench top and remote automation options will steadily increase the ubiquity of mechanisation in life science research. Such progressive adoption of automation will emphasise the already growing interdisciplinary nature of research further blurring the boundary between science and engineering.

## Author Contributions

IH conceived the study and wrote the manuscript with support from JD who critically reviewed it and also contributed content. Both authors contributed to the article and approved the submitted version.

## Conflict of Interest

The authors declare that the research was conducted in the absence of any commercial or financial relationships that could be construed as a potential conflict of interest. JD is a member of the Free Software Foundation, which campaigns for free (open-source) software in place of proprietary.
